# Impact of Orthodontic Forces on Plasma Levels of Markers of Bone Turnover and Inflammation in a Rat Model of Buccal Expansion

**DOI:** 10.3389/fphys.2021.637606

**Published:** 2021-05-25

**Authors:** Jan C. Danz, Alpdogan Kantarci, Michael M. Bornstein, Christos Katsaros, Andreas Stavropoulos

**Affiliations:** ^1^Department of Orthodontics and Dentofacial Orthopedics, School of Dental Medicine ZMK, University of Bern, Bern, Switzerland; ^2^The Forsyth Institute, Cambridge, MA, United States; ^3^Department of Oral Health and Medicine, University Center for Dental Medicine Basel UZB, University of Basel, Basel, Switzerland; ^4^Division of Regenerative Dental Medicine and Periodontology, University Clinics of Dental Medicine (CUMD), University of Geneva, Geneva, Switzerland; ^5^Department of Periodontology, Faculty of Odontology, Malmö University, Malmö, Sweden

**Keywords:** bone biology and physiology, orthodontic forces, orthodontic tooth movement, maxillary expansion, receptor activator of nuclear factor kappaB ligand, osteoprotegerin, parathyroid hormone, interleukin

## Abstract

Plasma levels of protein analytes might be markers to predict and monitor the kinetics of bone and tissue remodeling, including maximization of orthodontic treatment stability. They could help predict/prevent and/or diagnose possible adverse effects such as bone dehiscences, gingival recession, or root resorption. The objective of this study was to measure plasma levels of markers of bone turnover and inflammation during orthodontic force application in a rat model of orthodontic expansion. Two different orthodontic forces for bilateral buccal expansion of the maxillary arches around second and third molars were applied in 10 rats equally distributed in low-force (LF) or conventional force (CF) groups. Four rats served as the control group. Blood samples were collected at days 0, 1, 2, 3, 6, 13, 21, and 58. Longitudinal concentrations of osteoprotegerin (OPG), soluble receptor activator of nuclear factor kappaB ligand (sRANKL), interleukin-4 (IL-4), interleukin-6 (IL-6), interleukin-10 (IL-10), tumor necrosis factor α (TNF), and parathyroid hormone (PTH) were determined in blood samples by a multiplex immunoassay. CF and LF resulted in a significantly maxillary skeletal expansion while the CF group demonstrated significantly higher expansion than the LF group in the long term. Bone turnover demonstrated a two-phase response. During the “early phase” (up to 6 days of force application), LF resulted in more sRANKL expression and increased sRANKL/OPG ratio than the CF and control animals. There was a parallel increase in PTH levels in the early phase in response to LF. During the “late phase” (6–58 days), the markers of bone turnover were stable in both groups. IL-4, IL-6, and IL-10 levels did not significantly change the test groups throughout the study. These results suggest that maxillary expansion in response to different orthodontic forces follows different phases of bone turnover that may be force specific.

## Introduction

Orthodontic expansion of arches and facial tooth movement is frequently used to treat crowding and skeletal discrepancies (Weinberg and Sadowsky, 1996; Artun and Grobéty, 2001; Allais and Melsen, 2003). Optimal force application for orthodontic expansion of arches is critical as the arch expansion may be associated with unwanted effects such as gingival recession and bone loss (Batenhorst et al., 1974; Wennström et al., 1987; Wehrbein et al., 1994; Ackerman and Proffit, 1997; Joss-Vassalli et al., 2010). Thus, the magnitude of the orthodontic force may be important for a successful treatment outcome, although this is an understudied area in the context of buccal expansion. A limited number of studies have explored the optimal force magnitude (Danz et al., 2013; Utreja, 2018; Utreja et al., 2018) without a clear consensus on its impact on biological outcomes. There is also a prospect that the buccal expansion may not follow a phased process similar to the mesiodistal movement (Von Böhl et al., 2004). After force application, an initial phase of the movement is followed by the lag phase, wherein necrotic tissue is eliminated by undermining resorption. After that, continuous tooth movement occurs in the post-lag phase when direct bone resorption creates space ahead of the path of tooth movement. It is unclear whether a tissue response would follow the exact molecular mechanisms as the conventional orthodontic tooth movement in buccal expansion. The proximity of the periodontal ligament to the periosteum of the alveolar surface in expansive tooth movement may change biochemical regulation and tissue response.

Periodontal tissue remodeling in response to biomechanical forces is regulated by complex cellular mechanotransduction resulting from stress/strain on extracellular fiber strands and/or by paracrine factors and involves a non-infectious inflammatory process (Gruber et al., 1994; Roberts, 2004; Balce et al., 2011). Thus, it is plausible that the impact of buccal orthodontic tooth expansion and different levels of force application would potentially result in varying degrees of the release of inflammatory and bone turnover-associated markers. Tissue remodeling may be initiated by a cascade of pro-inflammatory cytokines such as interleukin-1 (IL-1), interleukin-6 (IL-6), and tumor necrosis factor α (TNF), which have been linked to wound healing during the acute phase of inflammation (Barber et al., 1999). This could be relevant since buccal orthodontic forces might cause tissue degradation in areas where the blood flow is interrupted during tooth movement inducing the development of local inflammation. Meanwhile, interleukin-4 (IL-4) has been shown to play an important role in tissue remodeling by activating macrophages and increasing their production of lysosomal enzymes for degradation of phagocytosed proteins (Balce et al., 2011) and by inducing increased collagen matrix production by fibroblasts (Bellini et al., 2012). Interleukin-10 (IL-10) is secreted by various cells (e.g., T cells), downregulates inflammation (Groux et al., 1997), and prevents scar tissue formation during wound healing (Kieran et al., 2013). For a well-orchestrated tissue response to the biomechanical forces without causing adverse effects on the periodontium, the impact of “pro-inflammatory” markers should be counter-regulated by the actions of “anti-inflammatory” cytokines. Overall, the balance between the inflammatory cytokines is critical for the tissue turnover in response to mechanical stimuli.

Inflammation regulates bone response, which can be monitored by measuring osteoblastic and osteoclastic activity markers. Receptor activator of nuclear factor kappaB (RANK), its ligands (RANKL and sRANKL), and osteoprotegerin (OPG) are critical players in the regulation of osteoclastogenesis and osteoblastogenesis during bone remodeling (Kenkre and Bassett, 2018). This pathway is dependent on a close cross-talk between the osteoblasts and osteoclasts. RANKL/RANK/OPG signaling induces osteoclast differentiation, fusion, activation, and survival. Soluble RANKL can be produced by osteoblasts, osteocytes, chondrocytes, and by gingival or periodontal fibroblasts. The osteocytes within the bone matrix respond to the changes in load and microdamage by soluble receptor activator of nuclear factor kappaB ligand (sRANKL) production and stimulate the initial osteoclastogenesis during bone remodeling (Xiong et al., 2013; Ono and Nakashima, 2018). OPG, a decoy receptor for RANKL, is secreted by osteoblasts and osteocytes, and inhibits osteoclastic bone resorption by binding to sRANKL, thus preventing its binding RANK. Therefore, the sRANKL/OPG ratio is key in regulating bone resorption, bone mass, and skeletal integrity and is modulated by several systemic factors, including parathyroid hormone (PTH, vitamin D, and inflammatory cytokines). Thus, as in inflammation, the balance between the anabolic and catabolic activities resulting from osteoblast and osteoclast function is critical for tissue restoration after the buccal orthodontic tooth movement.

In our previous work, we developed and characterized a rat model for orthodontic expansion to study the clinical and histological effects of two different forces (Danz et al., 2013, 2016). In the present study, the aim was to focus on the earlier expression of biological markers of bone turnover and inflammation and test the hypothesis that different levels of buccal orthodontic forces will result in varying degrees of impact on tissues and biological processes in different phases. Therefore, this experiment evaluated the time-dependent impact of two different orthodontic force application levels for buccal expansion on plasma levels of markers of inflammation (IL-4, IL-6, IL-10, and TNF) and bone metabolism markers (sRANKL, OPG, and PTH).

## Materials and Methods

### Animal Model and Experimental Design

**Figure 1:**
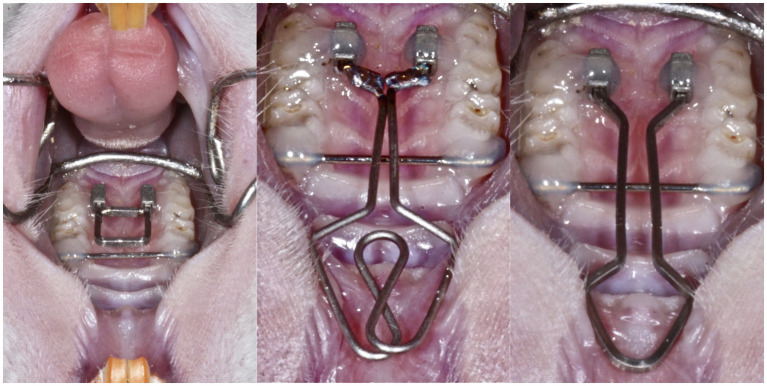
In this animal model, expansion forces were applied on the second and third molars: a low force (middle) and a conventional force (right). The cheek retractor (left) provided access for microscopic view on the operation field during the appliance placement. The transverse position of the first molars was stabilized with a transpalatal bar. Then, a guide was used to standardize the placement of the attachments for appliance insertion. The low-force spring was a 0.016" diameter round beta-titanium wire welded on 0.016"/0.022" ends. For the conventional force spring, a beta-titanium 0.016"/0.022" wire was used. The ends of the spring were fixated with composite in the tubes.

Fourteen male Wistar rats aged 15 weeks were used for blood sampling and analysis. The animals were kept pairwise in authorized plastic cages under controlled temperature, 12 h light cycle, and humidity with free access to standard food and tap water. Low- and conventional force springs were placed alternately as previously described in the animal model for buccal expansion (Danz et al., 2013, 2016). As shown in Figure 1, different magnitudes of force were applied to the second and third molars for expansion in this animal model: a low force (round spring, middle) and a conventional force (rectangular spring, right). The cheek retractor provided access for microscopic view on the operation field during the appliance placement. The transverse position of the first molars was stabilized with a transpalatal bar. Then, a guide was used to standardize the placement of the attachments for appliance insertion. The low-force spring was a 0.016" diameter round beta-titanium wire welded on 0.016"/0.022" ends. For the conventional force spring, a beta-titanium 0.016"/0.022" wire was used. The ends of the spring were fixated with composite in the tubes. Thus, transpalatal springs were used to apply a low-force (LF) or a conventional force (CF) bilaterally on the second and third maxillary molars (M2 and M3). A transpalatal bar was bonded to stabilize the first molar (M1). Animals in the conventional force group (CF; *n* = 5) received 23 cN/tooth, decreasing to 10 cN/tooth during deactivation, while animals in low-force group 2 (LF; *n* = 5) received 6 cN/tooth decreasing to 3 cN/tooth during deactivation. Four animals served as controls (group C). All appliances were inserted at baseline (Day 0) according to a standard etch-bond protocol (Danz et al., 2013). All animals were prepared for appliance placement by subcutaneous injection of 1 ml/kg of an anesthetic mixture [2.5 ml Hypnorm^®^ (Fentanyl citrate 0.315 mg/ml and fluanisone 10 mg/ml; Vetapharm Inc.), 2.5 ml Cepetor vet.^®^ (medetomidine hydrochloride 1.0 mg/ml; Scanvet Animal Health A/S), 0.5 ml Atropine DAK (atropine sulfate 1 mg/ml; Orifarm GmbH), and 4.5 ml distilled water]. The anesthesia duration was shortened after appliance placement by subcutaneous injection of 1 ml/kg of a mixture consisting of 2.5 ml Naloxone B. Braun^®^ (naloxone hydrochloride 0.4 mg/ml; B. Braun Melsungen Inc.), 4.0 ml Atipam^®^ (atipamezole hydrochloride 5.0 mg/ml; Eurovet Animal Health B.V.), and 3.5 ml distilled water (Danz et al., 2016). The Danish Inspectorate approved the study protocol for Animal Experiments (2010/561-1849).

### Impact of Buccal Expansion on Maxillary Skeletal Width

The maxillae were dissected free, dehydrated in ascending ethanol concentrations, and embedded in methacrylate for non-decalcified histology. The sections were obtained using a virtual cutting plane definition based on micro-CT registrations described in detail (Danz et al., 2014). Briefly, one non-decalcified section with the major palatine nerve was obtained and digitized with an Olympus BX51 microscope. Maxillary skeletal width was measured from the opening of the major palatine nerve to the mid-palatal suture on calibrated histomicrographs with the software Archimedes Geo3D (v1.3.6, Gottingen, Germany), as described (Danz et al., 2016).

### Blood Sampling and Analysis

To study the markers of inflammation and bone metabolism in the systemic circulation, 1 ml of blood was collected from the retro-orbital area (between 8 and 9 a.m.) on days 0, 1, 2, 3, 6, 13, 21, and 58, under full narcosis (isoflurane 1.5%, Forene^®^). The blood was then centrifuged at 2,000 rpm and 21°C for 10 min and again for 5 min after removing the supernatant, within 1 h from sampling, using a Sigma 1–15 K microfuge (Sigma Laborzentrifugen GmBH, Osterode am Harz, Germany). The fresh serum (about 0.6 ml from each sample) was stored at −80°C until analyses.

IL-6, IL-4, IL-10, TNF, PTH, sRANKL, and OPG levels were determined by multiplexed sandwich immunoassay (Bio-Rad, Hercules, CA, United States) using the flowmetric multiplex analysis. The antibody-coated beads were added into each well together with samples or standards and incubated for 2 h in the dark. The wells were washed with a vacuum manifold, and a detection antibody conjugated to biotin was added. After incubation for 1 h, beads were washed, followed by an incubation of 30 min with streptavidin conjugated to the fluorescent protein R-phycoerythrin (streptavidin-RPE). After washing the unbound streptavidin-RPE away, the beads were analyzed in the Luminex^™^100 (Luminex Corporation, Austin, TX, United States) to monitor spectral properties of the beads while simultaneously measuring the amount of fluorescence associated with R-phycoerythrin. Data were analyzed using Bio-Plex software (Luminex Corporation, Austin, TX, United States). Data were presented as concentration (pg/ml).

### Statistical Analysis

A multivariate repeated measurement analysis was used to test the influence of force and time and interactions on plasma levels of inflammation markers and bone turnover using Stata version 12.1 (StataCorp., College Station, United States).

## Results

### Buccal Expansion With Low and Conventional Orthodontic Forces

**Figure 2:**
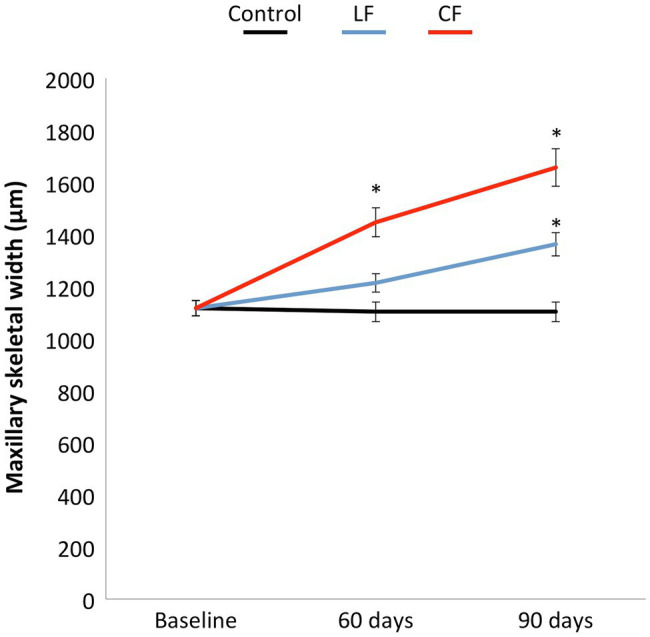
The low or conventional transpalatal continuous force created in addition to tooth movement a constant increase in maxillary skeletal width in both force groups. A larger opening of the midpalatal suture was found for the conventional force. The skeletal width in the control group remained stable.

The results of dental and skeletal effects of buccal expansion were published in Danz et al. (2016). The moved teeth were positioned more buccally with time in comparison with the teeth in the control group. Relevant to this study, CF resulted in a significant increase in skeletal width at the end of 60 days compared to baseline. The impact of LF was substantial, while not statistically significant compared to baseline by 60 days (Figure 2). Maxillary skeletal width increased insignificantly in the LF and CF groups compared to baseline at 90 days.

### Impact of Bilateral Buccal Expansion on Markers of Bone Turnover

**Figure 3:**
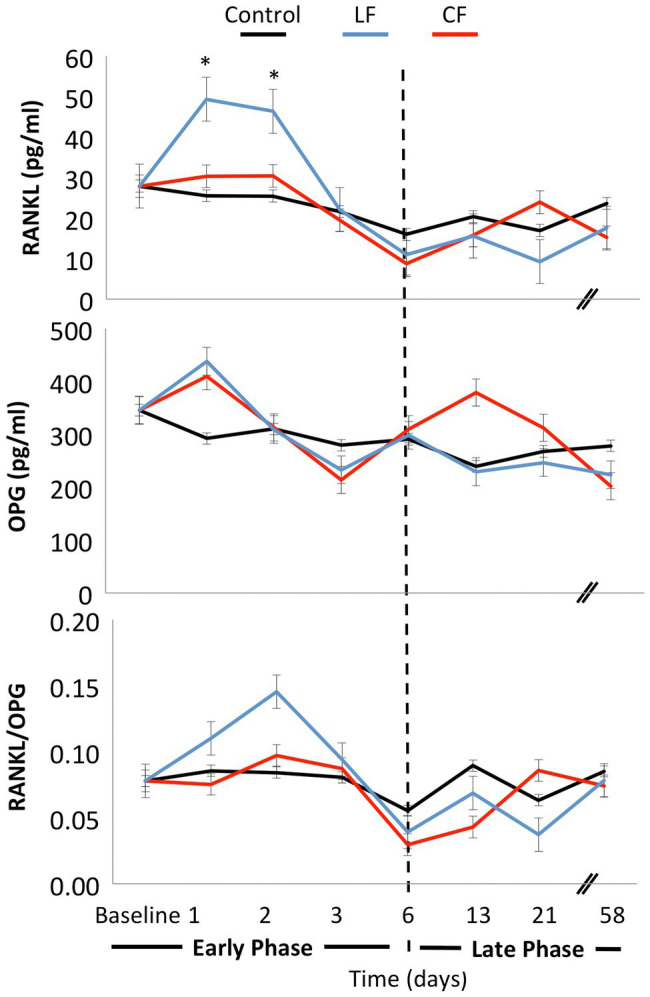
Mean concentrations of the bone markers OPG and sRANKL were measured at day 0, day 1, day 2, day 3, day 6, day 13, day 21, and day 58. The ratio sRANKL/OPG was calculated for each day. Both conventional and low force had an impact on sRANKL and OPG levels. In the early phase, a peak of soluble RANKL concentration was measured on days 1 and 2 in the low-force group. In the late phase from day 6 on, the levels stayed constant. No significant differences were measured for sRANKL/OPG concentrations over time or between force groups.

**Figure 4:**
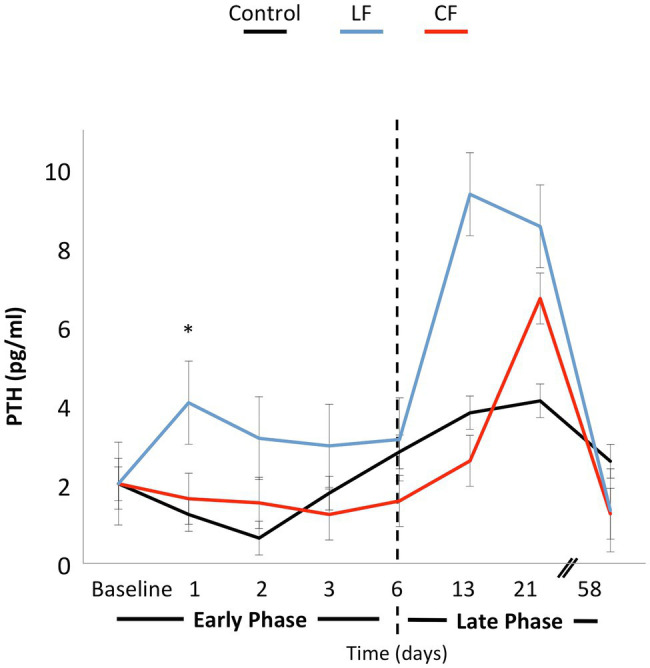
The line graph shows the variation of PTH concentrations over time. Force application did not alter the PTH level significantly. On the first and second days, PTH concentrations were elevated in the LF group. In the late phase, differences over time or between force groups were not significantly different.

The application of conventional orthodontic force had a significant impact on sRANKL levels (*p* = 0.03), while the impact of LF was borderline (*p* = 0.06). When looking at each time point, sRANKL levels were also elevated in the LF group during the “early phase” at days 1 and 2 (*p* < 0.01), but not anymore in the “late phase” after day 6. The sRANKL concentrations between only the two force levels were not significantly different (*p* = 0.76). OPG concentrations were lowered by applying conventional and low orthodontic forces (*p* < 0.01) without a significant difference between CF and LF. sRANKL/OPG ratio was not altered over time or by application of force (*p* = 0.21–0.86; Figure 3). An expansive force did not have a general influence on PTH levels. On the second day, during the “early phase,” PTH levels were elevated for LF (*p* < 0.01) and borderline for CF (*p* = 0.05; Figure 4).

### Impact of Bilateral Buccal Expansion on Plasma Levels of Markers of Inflammation

**Figure 5:**
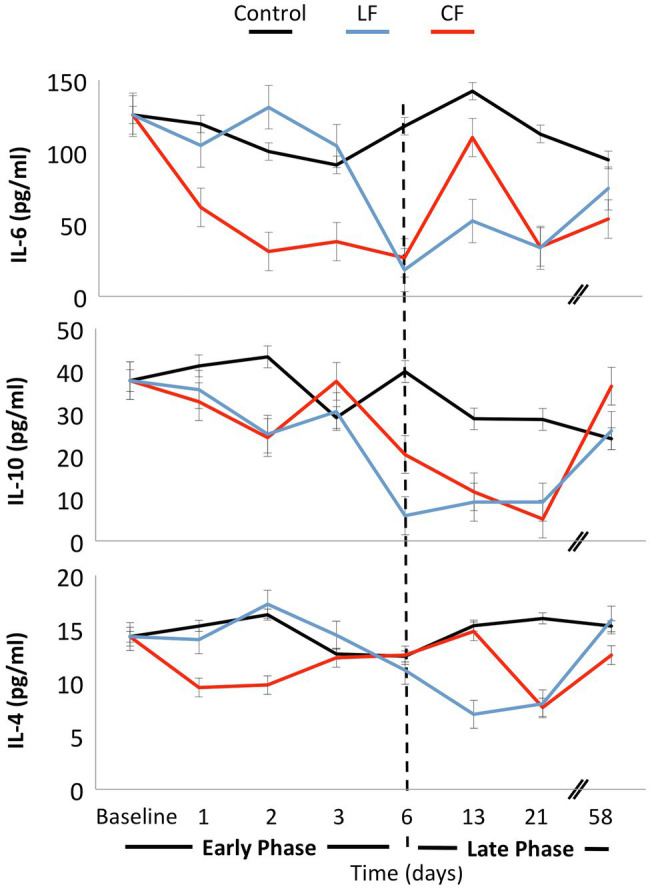
No significant fluctuations of the cytokines IL-4, IL-6, and IL-10 were found over time, and the application of force did not influence these inflammatory markers.

Neither force nor time showed a significant impact on IL-6, IL-4, or IL-10 levels in the serum (Figure 5). The concentration of TNF was below the detection limit of the assay for most of the samples.

## Discussion

In the present experimental study in rats, the aim was to investigate whether two different orthodontic force levels have a systemic impact on inflammatory (IL-4, IL-6, IL-10, and TNF) or bone markers (sRANKL, OPG, and PTH). Conventional orthodontic forces caused more skeletal widening of the maxillae than low forces. These findings are consistent with the larger skeletal width detected after rapid maxillary expansion than after slow maxillary expansion (Lo Giudice et al., 2017). Transpalatal application of low forces on molars had an impact on skeletal maxillary width in this study.

Conventional orthodontic tooth movement will migrate the teeth in the mesiodistal direction. Extensive research has been focused on this type of orthodontic tooth movement where the bone is resorbed ahead of the root during mesial tooth movement. On the other hand, there are not enough data on the impact of buccal expansion by the orthodontic forces. Traditionally, it was assumed that the tissue responses and the impact of buccal expansion would replicate the mesiodistal migration of the teeth while the anatomy and resistance to force are different. The buccal movement against the cortical wall also involves apposition on the periosteal surface (Kraus et al., 2014). Therefore, the tissue regulation must differ between the two types of movements, one leading to resorption followed by apposition while the other leading to apposition as a partial adaptation.

To date, there are not much data available about the influence of orthodontic force application on serum values of sRANKL or OPG. However, it seems that orthodontic tooth movement causes changes in systemic sRANKL and OPG concentrations. Possible explanations of the source of increased sRANKL would be from necrotic tissue and resulting proteolysis of transmembrane receptors or upregulation of primary secretion (Rogers, 2005). In this study, we used the sRANKL and OPG as indicators for bone resorption and turnover in line with a previous publication (Ferreira et al., 2016). Further studies are needed to identify the source of sRANKL. It is known that the soluble forms of RANKL detected in serum are generated by proteolysis of transmembrane proteins or by alternative mRNA splicing (Hughes and Crispe, 1995) and circulate in the blood partially bound to ligands. However, their physiological activity – particularly in bone metabolism – and their stability are not fully known (Simonet et al., 1997; Furuya et al., 2001; Findlay and Atkins, 2011).

The sRANKL/OPG ratio was found to be changed by periodontal repair at tension sides after tooth movement (Lossdörfer et al., 2010). The constant sRANKL/OPG ratio herein can be interpreted as the normal equilibrium of coupled bone homeostasis between bone resorption and bone formation during tooth movement with constant forces.

Another possibility is that local changes in sRANKL/OPG ratio were not extensive enough to be detected in the blood serum or were masked by normal bone turnover in the orbital region. Elevated PTH levels at day 2 and a trend for PTH levels to fluctuate also in the late phase may be a response to low calcium levels from initiation of bone apposition at the intermaxillary suture, and therefore, tooth movement may be modulated by PTH (Lossdörfer et al., 2010; Blaine et al., 2015).

The deactivation of the appliance was minimal in the first days, and at day 57, the force reduction was approximately 20–30% *in vitro*. While this may be a confounding factor for the long-term responses in the rat model, exertion of continuous forces in this study would compensate for any variation over time.

The sampling timing was chosen, assuming that inflammatory marker values would vary mainly during the initial days and level back and stabilize after the first week (Schröder et al., 2018). In later stages of force application, local tissues have adapted to the force application, and systemic cytokines may not be altered anymore, which in turn may explain the normal systemic levels of C-reactive protein, TNF, and IL-6 reported after 2, 4, and 6 months of headgear treatment (MacLaine et al., 2010). IL-4 and IL-10 have been reported to inhibit the secretion of macrophage colony-stimulating factor and decrease survival and differentiation of mononuclear phagocytes (Gruber et al., 1994). IL-4 can act as a chemotactic and differentiating factor for osteoblasts (Lind et al., 1995) and as a suppressor of osteoclast development and function (Moreno et al., 2003). In an intermediate phase, hypothetically, a systemic presence of IL-4 could be expected after a few days of orthodontic force application, when tissue is repaired and remodeled. A significant overall impact of force and time on IL-4, IL-6, and IL-10 blood serum was not detected in the present study. This might be attributed to the limited number of animals. On the other hand, a clear cascade of pro- and anti-inflammatory markers known from fracture healing (Kon et al., 2001; Pape et al., 2010) might not be present during orthodontic force application. If low forces are used, the second phase of tooth movement with the elimination of necrotic tissues is skipped, and thus, the inflammatory reaction is lower or disappears completely (Kohno et al., 2002; Von Böhl et al., 2004).

The results in blood serum presented herein are partially consistent with studies on biomarkers in saliva: No alteration in the levels of the biomarker of inflammation or bone turnover was found in saliva at monthly intervals during orthodontic tooth movement (Reiss et al., 2020). When observing shorter periods, the most significant increase in IL-1β was found 1 day after the insertion of a separation ligature in the gingival crevicular fluid specimen (Dudic et al., 2006). Salivary bone markers were reported to be unchanged, or a time-related increase in sRANKL, decreased OPG, and the changes in sRANKL/OPG ratio were measured during 8 weeks (Florez-Moreno et al., 2013; Reiss et al., 2020). This study suggests a release of sRANKL during an early phase response after orthodontic force application.

While the work provides a basis for using the serum analytes as markers of change at the tissue level, the overarching goal was not to “discover biomarkers.” We aimed to identify the systemic changes in response to orthodontic force application. As this is the only intervention in the animals, the systemic changes are associated directly with the magnitude of the force and orthodontic expansion of the jaw. This is a highly novel and previously unknown result. Interestingly, the pattern that is observed in this model is not following the conventional orthodontic tooth movement. Therefore, we think the data represent a novel biological outcome, and the clinical relevance of this model is high. In humans, the application of orthodontic force is described by the patient as a feeling of pain similar to that in an inflammatory process. “The patient feels a mild aching sensation, and the teeth are sensitive to pressure so that biting a hard object hurts. Such pain typically and reportedly lasts 2–4 days and then disappears until the orthodontic appliance is reactivated. A similar cycle may recur at that point, but for almost all patients, the pain associated with the initial activation of the appliance is the most severe” (Proffit and Fields, 2012). Thus, the inflammatory process described in this work represents a systemic outcome of local tissue response.

The analysis of biomarkers in the serum and other biological fluids in man and animals is an emerging field in understanding the regulations of biological processes. Although biologically relevant cytokines act locally in the tissue, systemic serum markers might help study and assess the kinetics of bone turnover under physiological and pathological conditions. Furthermore, the knowledge of the levels could help prevent or diagnose, at an early stage, possible adverse effects of facial orthodontic tooth movement such as bone dehiscences, gingival recession or root resorption, and maximization stability of the treatment outcome. Patients prone to adverse effects may show abnormal inflammatory or bone metabolism values before they become clinically apparent. Further research is needed to validate levels of systemic inflammatory and bone metabolism markers as an indicator for local physiological or pathological conditions during orthodontic tooth movement. An important technical limitation of this study is the lack of a universally accepted and validated measurement technique. This could explain some variation and could confound some potentially statistically significant results (Fu et al., 2010).

## Conclusion

This study’s findings suggested that the buccal expansion may result in a different tissue response than the mesiodistal orthodontic tooth movement at different phases of healing and independent of force.

## Data Availability Statement

The raw data supporting the conclusions of this article will be made available by the authors, without undue reservation.

## Ethics Statement

The animal study was reviewed and approved by Danish Inspectorate for Animal Experiments (2010/561-1849).

## Author Contributions

JD involved in animal experiments, statistics, and manuscript preparation. AK involved in blood analysis, graphs, and manuscript preparation. MB wrote and revised the manuscript. CK oversaw the project. AS oversaw the project and was involved in blood sampling and revision. All authors contributed to the article and approved the submitted version.

### Conflict of Interest

The authors declare that the research was conducted in the absence of any commercial or financial relationships that could be construed as a potential conflict of interest.
